# *ABO* rs657152 and Blood Groups Are as Predictor Factors of COVID-19 Mortality in the Iranian Population

**DOI:** 10.1155/2022/5988976

**Published:** 2022-11-14

**Authors:** Fahimeh Mirzaei Gheinari, Fatemeh Sakhaee, Melika Gholami, Fattah Sotoodehnejadnematalahi, Mohammad Saber Zamani, Iraj Ahmadi, Enayat Anvari, Abolfazl Fateh

**Affiliations:** ^1^Department of Biology, Science and Research Branch, Islamic Azad University, Tehran, Iran; ^2^Department of Mycobacteriology and Pulmonary Research, Pasteur Institute of Iran, Tehran, Iran; ^3^Immunoregulation Research Center, Shahed University, Tehran, Iran; ^4^Department of Physiology, School of Medicine, Ilam University of Medical Science, Ilam, Iran; ^5^Microbiology Research Center (MRC), Pasteur Institute of Iran, Tehran, Iran

## Abstract

Several studies have discovered a relationship between specific blood types, genetic variations of the *ABO* gene, and coronavirus disease 2019 (COVID-19). Therefore, the aim of this study was to evaluate the association between *ABO* rs657152 polymorphisms and ABO blood groups with COVID-19 mortality. The tetraprimer amplification refractory mutation system, polymerase chain reaction method, was used for *ABO* rs657152 polymorphism genotyping in 1,211 dead and 1,442 improved patients. In the current study, the frequency of *ABO* rs657152 AA than CC genotypes was significantly higher in dead patients than in improved patients. Our findings indicated that blood type A was associated with the highest risk of COVID-19 mortality compared to other blood groups, and patients with blood type O have a lower risk of infection, suggesting that blood type O may be a protective factor against COVID-19 mortality. Multivariate logistic regression test indicated that higher COVID-19 mortality rates were linked with alkaline phosphatase, alanine aminotransferase, high density lipoprotein, low-density lipoprotein, fasting blood glucose, uric acid, creatinine, erythrocyte sedimentation rate, C-reactive protein, 25-hydroxyvitamin D, real-time PCR Ct values, ABO blood groups, and *ABO* rs657152 AA genotype. In conclusion, the AA genotype of *ABO* rs657152 and blood type A were associated with a considerably increased frequency of COVID-19 mortality. Further research is necessary to validate the obtained results.

## 1. Introduction

Coronavirus disease 2019 (COVID-19) is a global pandemic caused by Severe Acute Respiratory Syndrome coronavirus 2 (SARS-CoV-2) [1]. COVID-19 has become a global issue due to its simplicity of transmission and rapid spread via close contact. Since the disease is contagious during the incubation period, controlling the spread of this pandemic is extremely difficult. Furthermore, the current study found that 10%-30% of COVID-19 subjects were asymptomatically and could easily become a viral transmission conduit [2].

In general, risk factors are variables correlated with a greater likelihood of contracting an infection. Nonmodifiable risk factors and modifiable risk factors are the 2 categories of risk factors [3]. The ABO blood type is a hereditary trait that cannot be changed. Individuals can be classified as A, B, AB, or O based on the presence or absence of antigens on erythrocyte surfaces. Several studies have revealed relationships between ABO blood types and viral respiratory diseases such as influenza A (H1N1) and SARS [4, 5]. Numerous studies have recently hypothesized associations between blood types and susceptibility to COVID-19, its role in the disease's progression and its consequences [6, 7].

To find host genetic variables linked to the progression of SARS-CoV-2 infections, genome-wide association studies (GWAS) were carried out by COVID-19 Host Genetics Initiative (HGI) [8]. Numerous single-nucleotide polymorphisms (SNPs) and genes were shown to be linked to infection susceptibility or other characteristics of disease severity, such as the need for hospitalization, respiratory failure, or mortality [9].

The human ABO blood group gene is found on chromosome 9, which is linked to the production of certain ABO glycosyltransferases [10]. One study found that analyzing SNP rs657152 (which is located in the ABO gene's intron area of the *ABO* gene (chr 9q34.2) and is a factor in determining blood types) could determine the severity of an illness [11]. In light of this context, we investigated whether the previously reported associations between the variants of rs657152 in the *ABO* gene and SARS-CoV-2 infection could also be observed in an Iranian cohort with SARS-CoV-2 infection.

## 2. Material and Methods

### 2.1. Study Participants

The current study was approved by the Ethics Committee of the Pasteur Institute of Iran (PII) (IR PII REC 1400.042). Informed written consent was obtained from all patients according to the 1975 Declaration of Helsinki and applicable local rules.

COVID-19 patients were recruited from PII from June 2021 to February 2022. A total of 2,653 patients who tested positive for SARS-CoV-2 using reverse transcriptase real-time polymerase chain reaction (rtReal Time-PCR) were enrolled in this cohort study. The patients with pulmonary disease including chronic obstructive pulmonary disease, cystic fibrosis, asthma, lung cancer, pregnancy, heart disease, liver disease, chronic kidney disease, diabetes, obesity, and human immunodeficiency virus (HIV) were excluded in the study.

The laboratory parameters, such as triiodothyronine (T3), thyroxine (T4), thyroid-stimulating hormone (TSH), uric acid, serum creatinine, erythrocyte sedimentation rate (ESR), C-reactive protein (CRP), white blood cells (WBC), platelets, fasting blood glucose (FBS), 25-hydroxyvitamin D, triglyceride (TG), high density lipoprotein (HDL), low-density lipoprotein (LDL), cholesterol, alkaline phosphatase (ALP), aspartate aminotransferase (AST), alanine aminotransferase (ALT), real-time PCR cycle threshold (Ct) values, and ABO blood groups were extracted from the patients' medical records.

### 2.2. DNA Extraction and *ABO* rs657152 Genotyping

A total of 10mL of blood was obtained from each patient. Using Ficoll (Ficoll-Paque PLUS, GE Healthcare, USA) density gradient centrifugation, peripheral blood mononuclear cells (PBMCs) were separated from the samples and stored at -70°C. The genomic DNA from PBMCs was extracted using the High Pure PCR Template Preparation Kit (Roche Diagnostics Deutschland GmbH, Mannheim, Germany), according to the manufacturer's instructions. The tetraprimer amplification refractory mutation system PCR (T-ARMS-PCR) method was used to genotype *ABO* rs657152.

The PRIMER1 website was used to design the primers for *ABO* rs657152 (http://primer1.soton.ac.uk/primer1.html). The oligo-analyzer was then used to check the primer sequences for heterodimers and hairpins. The mismatched nucleotide was added at the third position from the 3′-end terminal of the primers to facilitate a reliable differentiation between the 2 alleles (Table 1).

PCR was performed in a 25-*μ*L reaction volume with 15 *μ*L of TEMPase Hot Start DNA Polymerase (Ampliqon, Hamburg, Germany), 60 ng of extracted DNA, 4.0 *μ*L of 5.0pmol of the outer forward primer, 3.0 *μ*L of 5.0 pmol of the outer reverse primer, 4.0 *μ*L of 10 pmol of the inner forward primer, and 4.0 *μ*L of 10 pmol of the inner reverse primer. PCR was carried out using the initial denaturation at 95°C for 20 minutes, followed by 42 cycles of 95°C for 35 seconds, 55°C for 30 seconds, and 72°C for 30 seconds, with a final extension at 72°C for 10 minutes. The PCR results were visualized by electrophoresis on a 3% agarose gel. The result of *ABO* rs657152 genotyping with T-ARMS-PCR has illustrated in Supplementary Figure 1.

DNA sequencing was performed by the ABI 3500 DX Genetic Analyzer (ABI, Thermo Fisher Scientific, Waltham, MA, USA) to confirm the T-ARMS-PCR results. MEGA version 11.0 was used to assess the raw sequencing data (https://www.megasoftware.net/). The sequencing results of *ABO* rs657152 genotypes for confirming the T-ARMS-PCR method has illustrated in Supplementary Figure 2.

### 2.3. Statistical Analysis

The following analyses were performed by SPSS version 25 (SPSS Inc., Chicago, Ill., USA). The Pearson's chi-square test was used to assess the distribution of categorical variables. In this study, continuous variables were analyzed using the Mann–Whitney *U* test. Using multivariate logistic regression models, the association between *ABO* rs657152 and the probability of developing a COVID-19 infection was estimated. Odds ratios (ORs) and 95% confidence intervals (CIs) were generated to assess the strength of this association. A two-tailed *P* value <0.05 was regarded as statistically significant. The area under the curve-receiver operating characteristic (AUC-ROC) analysis was used to assess the effect of *ABO* rs657152 on the mortality of COVID-19 subjects. The correlation between COVID-19 mortality and *ABO* rs657152 was evaluated in codominant, dominant, recessive, and overdominant inheritance models by the SNPStats web software available at https://snapstat.net/snpstats/. The Akaike information criterion (AIC) and Bayesian information criterion (BIC) were used to select the best fit model for rs657152. HWE was assessed by the chi-square test (https://snapstat.net/snpstats/).

The Akaike information criterion (AIC) and the Bayesian information criterion (BIC) were used to select the best fit model for *ABO* rs657152. Hardy-Weinberg equilibrium (HWE) was assessed by the chi-square test.

## 3. Results

### 3.1. General Characteristics of the Studied Patients

Table 2 indicates the demographic data of COVID-19 patients. A total of 2,653 PCR-positive patients with COVID-19 were classified into 2 groups: dead (*n* = 1211) and improved (*n* = 1442) patients. The mean (±SD) age differed significantly between groups (dead patients have a higher mean age than improved patients) (58.1 ± 11.3 vs. 51.9 ± 12.8 years). Of 2,653 patients, 1400 (52.8%) and 1253 (47.2%) were male and female, respectively.

Mortality rates were associated with mean (±SD) age (*P* < 0.001), increased levels of ALT (*P* < 0.001), AST (*P* < 0.001), ALP (*P* < 0.001), ESR (*P* < 0.001), CRP (*P* < 0.001), FBS (*P* < 0.001), and Cr (*P* < 0.001), and decreased levels of 25-hydroxyvitamin D (*P* < 0.001), real-time PCR Ct value (*P* = 0.001), uric acid (*P* < 0.001), TG (*P* = 0.001), cholesterol (*P* < 0.001), LDL (*P* < 0.001), and HDL (*P* < 0.001).

### 3.2. Relationship between *ABO* rs657152, Blood Groups, and COVID-19 Mortality

**Figure 1:**
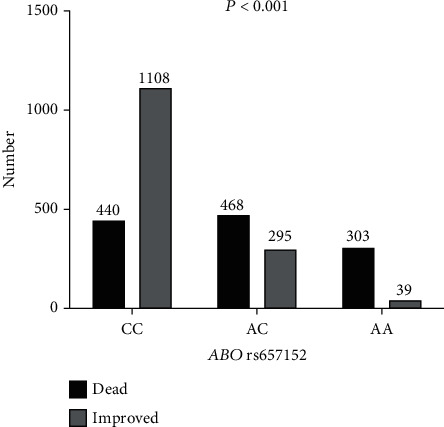
Frequency of *ABO* rs657152 in COVID-19 patients.

Figure 1 illustrates the relationship between *ABO* rs657152 and COVID-19 infection mortality. The patients with *ABO* rs657152 AA genotypes revealed significantly higher in dead patients compared to improved individuals; nevertheless, improved COVID-19 patients had CC genotypes (Figure 1).

**Figure 2:**
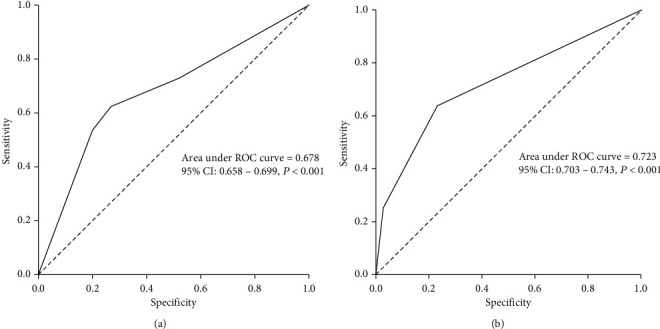
(a) ROC curve with the ABO blood groups. (b) *ABO* rs657152 for prediction the mortality rate in COVID-19.

SNPStats software was used to examine the *ABO* rs657152 inheritance model, which included codominant, dominant, recessive, and overdominant. For *ABO* rs657152, codominant with the lowest AIC and BIC values was the best-fitting inheritance model. A higher risk of death was linked to the *ABO* rs657152 AA genotype (OR 19.56, 95% CI 13.77-27.80, *P* < 0.001). *ABO* rs657152 genotypes were incompatible with HWE in all patients (*P* = 0.041). It should be considered that the HWE might not be met in the case sample, which may indicate that the SNP is related with the disease. Minor allele frequency (MAF) (A-allele) in dead, improved, and all patients was 0.44, 0.13, and 0.27, respectively (Table 3). In addition, the AUC-ROC values for *ABO* rs657152 were 0.723, showing that host genetic factors play a role in viral infection mortality (Figure 2(b)).

The distribution of ABO blood groups was statistically different between the 2 groups (*P* < 0.001). The dead patients had the blood groups of A (48.3%), B (24.9%), O (20.4%), and AB (6.4%), respectively (Table 2). Our finding indicated that blood type A was associated with the highest risk of COVID-19 mortality compared to other blood groups, and patients with blood type O have a lower risk of infection, suggesting that blood type O may be a protective factor against COVID-19 mortality. Furthermore, the AUC-ROC values for blood groups were 0.678, indicating that blood groups especially type A could play a role in COVID-19 mortality (Figure 2(a)).

### 3.3. Association between Risk Factors with COVID-19 Infection Mortality

The multivariate logistic regression model was used to assess the risk factors related to COVID-19 mortality. The COVID-19 mortality was associated with mean age (OR 0.967, 95% CI 0.956-0.978, *P* < 0.001), ALP (OR 0.997, 95% CI 0.996-0.999, *P* = 0.003), ALT (OR 0.984, 95% CI 0.977-0.990, *P* < 0.001), HDL (OR 1.035, 95% CI 1.022-1.047, *P* < 0.001), LDL (OR 1.018, 95% CI 1.015-1.022, *P* < 0.001), FBS (OR 0.996, 95% CI 0.993-0.999, *P* = 0.010), uric acid (OR 1.933, 95% CI 1.762-2.120, *P* < 0.001), creatinine (OR 0.089, 95% CI 0.058-0.136, *P* < 0.001), ESR (OR 0.971, 95% CI 0.962-0.980, *P* < 0.001), CRP (OR 0.980, 95% CI 0.974-0.987, *P* = 0.004), 25-hydroxyvitamin D (OR 1.040, 95% CI 1.028-1.052, *P* < 0.001), real-time PCR Ct values (OR 0.982, 95% CI 0.963-0.998, *P* = 0.041), ABO blood groups (OR 1.293, 95% CI 1.169-1.431, *P* < 0.001), and *ABO* rs657152 AA (OR 0.195, 95% CI 0.155-0.247, *P* < 0.001) (Table 4).

## 4. Discussion

This study is a comprehensive study to identify a strong association between ABO blood groups and the allele frequency of *ABO* rs657152 and COVID-19 mortality in Iran.

In this study, individuals with blood group A had a higher chance of COVID-19 mortality; whereas, patients with blood group O were somewhat protected against infection. Zhao et al. were the first to report the effect of the ABO blood type system on COVID-19 susceptibility in 3 separate hospitals in China. It was indicated that individuals with blood group A had a greater COVID-19 infection rate than blood group O, particularly in a location where the prevalence of blood categories A and O is 31% and 34% of the population, respectively. These results imply that blood group O may provide protection against infection, but blood group A makes individuals more susceptible to COVID-19 [12].

In accordance with our data, investigations from Iran, Iraq, Turkey, Denmark, and Lebanon indicated that blood group A had the largest proportion of COVID-19-positive cases and mortality than the other blood groups, and that the O blood group had the lowest number of infected individuals [13–17]. In contrast, a number of studies carried out in a variety of nations found no connection between the ABO blood groups and the severity of COVID-19 or the mortality rate [18, 19]. In a metaregression analysis of 101 nations with known populational blood group distributions, the study evaluated the data from 9 million COVID-19 patients with 450,000 deaths. After adjusting for 14 potential confounders, such as hypertension, obesity prevalence, and life expectancy at birth, the study found no association between groups A or B and overall mortality. However, group O was related to decreased mortality [20]. Nonetheless, these results are not conclusive. These contradictory results could be related to varied populations and their geographical regions, comparative controls chosen, and the presence of confounding variables (such as comorbidities), which some investigations did not adjust for. Another aspect that could explain the disparities in results is that some of the studies included volunteers chosen randomly as controls [21].

The hypothesis of a relationship between ABO blood groups and the severity of COVID-19 infection can be explained by several mechanisms, including anti-A antibodies found naturally in blood group O; individuals inhibit infection by binding to A-like antigens found on the SARS-CoV-2 envelope; also, blocking the connection between the S protein with the angiotensin-convertor enzyme-2 (ACE-2) receptor may inhibit viral entry into the lung epithelium; individuals with blood group A have higher ACE-1 activity (which could lead to a worsening of COVID-19 symptoms), glycan antigens production by SARS-CoV-2, and genetic polymorphisms in the ABO gene [22].

The ABO gene, which is located on chromosome 9, determines blood type. It has 7 exons and codes for enzyme glycosyltransferases and is involved in the production of antigens in blood types A and B [23].

In a meta-analysis, researchers examined 8,582,968 SNPs in 1,980 patients with COVID-19-related respiratory failure at 7 hospitals in Italy and Spain. Researchers discovered a cross-replicating connection with rs11385942 at 3p21.31 and rs657152 at 9q34. The 3p21.31 gene cluster was discovered to be a genetic susceptibility locus in COVID-19 patients with respiratory failure [24]. Using transethnic genome-wide association analyses, Shelton et al. also found a significant relationship between the blood type and COVID-19. Particularly, a locus on chr3p21.31 was strongly associated with outcome severity [25].

In this study, the *ABO* rs657152 AA genotype had a strong association with COVID-19 mortality. In line with our findings, several studies have shown that the *ABO* rs657152 A allele is significantly correlated with COVID-19 severity [11, 26], but in the previous 2 studies, this relationship was not shown [27, 28]. A study in Russia indicated that the *ABO* rs657152 distribution was more homogeneous. The association between mortality rate of COVID-19 and the frequency of *ABO* rs657152 was significant. They stated that these reasonable relationships were only found in the “Russian” dataset: no such associations were found in the “global” dataset. This could be due to discrepancies in the methodology used to collect COVID-19 statistics across nations [29]. The MAF of the risk allele (A) at *ABO* rs657152 in the current study was 0.27 in all patients and 0.44 in dead patients. The MAF for *ABO* rs657152 in 0.25 in Indonesians (0.48), South Koreans (0.48), European (0.37), African (0.49), Asian (0.43), other Asian (0.39), and Latin American (0.41) was reported in dbSNP the NCBI dbSNP database (https://www.ncbi.nlm.nih.gov/SNP/). This locus was discovered to be related with respiratory failure in European patients [11]. The pathophysiology of severe Covid-19 and the related respiratory failure is unknown, but greater age, male gender, diabetes, cardiovascular disease, and host genetic factors are consistently associated with higher mortality. However, the inappropriate and delayed management of emergency medical needs increased COVID-19 mortality during the pandemic. While the pandemic has been devastating for wealthier countries such as the United States and the United Kingdom, the world's poorest countries have been impacted the hardest, with women and children suffering a disproportionate share of the burden. Due to lack of access to medicine, proper equipment, and reporting, a considerable percentage of COVID-19-related deaths go unreported, particularly in the poorest nations. It seems that different access to disease treatment and/or clinical and hospital facilities in different countries can also affect these assessed endpoints [30–32].

In a study including 7,241 white and 1,671 black participants, *ABO* rs657152 was found to be related to 84 and 24 proteins, respectively. Seven of the 84 proteins found in the primary analysis, including ephrin type-A receptor 4 and von Willebrand factor type A, were substantially linked to the incidence of hospitalized pneumonia and respiratory infections [33].

Before being found to be a risk factor for severe COVID-19, rs657152 in the ABO blood locus group was linked to a hypercoagulable state, arterial embolism/thrombosis, and other circulatory system problems [34]. Also, *ABO* rs657152 correlates with an increased likelihood of hypercholesterolemia, diabetes, and heart failure as well as decreased odds of gastrointestinal diseases such as duodenal ulcer and duodenitis [35]. The genetic predisposition for these endocrine and cardiovascular phenotypes may increase the chance of adverse outcomes of COVID-19, but it may have longer-term health consequences [36]. Taken together, these correlations add support to risk factors that contribute to a hypercoagulable condition, as both COVID-19 infection and the *ABO* rs657152 risk allele itself may increase thrombosis risk through numerous routes [37].

Understanding the role of ABO and its relationship with COVID-19 severe manifestations requires significant effort. The genetic and serological evidence of the involvement of ABO blood types and *ABO* gene allelic relationships with COVID-19 severity gives a unique opportunity to explore the contribution of host genetics to interindividual phenotypic variation [38].

Based on previous studies and in line with our study, age, liver enzymes profiles, lipid profile, low 25-hydroxyvitamin D, uric acid, creatinine, and real-time PCR Ct values were associated with COVID-19 mortality [39–42].

In conclusion, according to these findings, blood types and *ABO* rs657152 may be risk factors for COVID-19 infection and disease severity in the Iranian population. Future studies will look into how genomic data might be linked to electronic health records to improve clinical management and patient outcomes. However, further investigation of these findings is necessary, both in terms of their utility in clinical risk profiling of COVID-19 patients and toward a molecular understanding of the underlying pathophysiology.

## Figures and Tables

**Table 1:** Sequences and melting temperatures of the *ABO* rs657152 primers.

SNP	Primer	Primer sequence	Size, bp	T_m_	Annealing temp
*ABO* rs657152 (A/C)	OF	5′-ACTACTTCATCTGTTACTTCTTATCTTAT-3′	—	61.0°C	55.0°C
	OR	5′-GTGTGAAACTCATCAAAACCGTATA-3′	339	61.0°C	
	IF	5′-TATCTCGAATAGCTTCTTGAAGC**C**-3′	255	61.8°C	
	IR	5′-TTGCCTCCCACGTTCCT-3′	124	62.0°C	

The specific nucleotides at 3′-end of primers are in bold, while the mismatches are underlined. SNP: single nucleotide polymorphism; OF: outer forward primer; OR: outer reverse primer; IF: inner forward primer; IR: inner reverse primer.

**Table 2:** Comparison laboratory parameters between dead and improved patients infected with COVID-19.

Variables	Dead patients (*n* = 1211)	Improved patients (*n* = 1442)	*P* value
Mean age ± SD	58.1 ± 11.3	51.9 ± 12.8	<0.001^∗^
Gender (male/female)	646/565 (53.3/46.7%)	754/688 (52.3/47.7%)	0.587
ALT, IU/L (mean ± SD) (reference range: 5-40)	44.6 ± 24.5	33.3 ± 24.5	<0.001^∗^
AST, IU/L (mean ± SD) (reference range: 5-40)	37.1 ± 13.2	31.2 ± 15.5	<0.001^∗^
ALP, IU/L (mean ± SD) (reference range: up to 306)	202.3 ± 66.0	167.9 ± 89.4	<0.001^∗^
Cholesterol, mg/dL (mean ± SD) (reference range: 50-200)	116.7 ± 39.7	122.6 ± 36.2	<0.001^∗^
TG, mg/dL (mean ± SD) (reference range: 60-165)	116.1 ± 41.3	132.3 ± 62.1	<0.001^∗^
LDL, mg/dL (mean ± SD) (reference range: up to 150)	69.5 ± 35.0	108.2 ± 49.2	<0.001^∗^
HDL, mg/dL (mean ± SD) (reference range: >40)	30.6 ± 10.9	34.4 ± 11.6	<0.001^∗^
WBC, 10^9^/L (mean ± SD) (reference range: 4000-10000)	7540.8 ± 2643.8	7732.8 ± 2897.6	0.263
CRP, mg/L (mean ± SD) (reference range: <10 mg/L negative)	67.7 ± 21.4	56.9 ± 20.8	<0.001^∗^
ESR, mm/1st h (mean ± SD) (reference range: 0-15)	55.4 ± 15.4	46.6 ± 15.4	<0.001^∗^
FBS, mg/dL (mean ± SD) (reference range: 70-100)	111.1 ± 43.2	105.8 ± 41.6	<0.001^∗^
Platelets ×1000/cumm (mean ± SD) (reference range: 140000-400000)	185 ± 77	184 ± 67	0.310
Uric acid, mg/dL (mean ± SD) (reference range: 3.6-6.8)	3.7 ± 1.2	5.7 ± 1.6	<0.001^∗^
Creatinine, mg/dL (mean ± SD) (reference range: 0.6-1.4)	1.1 ± 0.3	0.8 ± 0.3	<0.001^∗^
T3, ng/dL (mean ± SD) (reference range: 2.3-4.2)	3.2 ± 1.1	2.8 ± 0.7	0.052
T4, mcg/dL (mean ± SD) (reference range: 5.6-13.7)	9.0 ± 4.3	8.3 ± 3.6	0.156
TSH, mu/L (mean ± SD) (reference range: 0.4-4.5)	3.2 ± 1.5	3.1 ± 1.4	0.359
25-hydroxy vitamin D, ng/mL (mean ± SD) (sufficiency: 21-150)	26.9 ± 10.1	36.0 ± 13.6	<0.001^∗^
Real-time PCR Ct values	12.1 ± 6.4	27.1 ± 7.9	0.001^∗^
ABO blood groups			<0.001^∗^
A (%)	584 (48.3%)	390 (27.1%)	
B (%)	302 (24.9%)	155 (10.7%)	
AB (%)	78 (6.4%)	115 (8.0%)	
O (%)	247 (20.4%)	782 (54.2%)	

ALT: alanine aminotransferase; AST: aspartate aminotransferase; ALP: alkaline phosphatase; TG: triglyceride; LDL: low density lipoprotein; HDL: high density lipoprotein; WBC: white blood cells; CRP: C-reactive protein; ESR: erythrocyte sedimentation rate; FBS: fasting blood glucose; T3: triiodothyronine; T4: thyroxine; TSH: thyroid-stimulating hormone; Ct: cycle threshold; SD: standard deviation. ^∗^Statistically significant (<0.05).

**Table 3:** *ABO* rs657152 association with COVID-19 mortality.

Model	Genotype	Groups		OR (95% CI)	*P* value	AIC	BIC
		Improved patients	Dead patients				
Allele	C	2511 (73.0%)	1348 (56.0%)	—	—	—	—
	A	373 (27.0%)	1074 (44.0%)	—	—	—	—

Codominant	C/C	1108 (76.8%)	440 (36.3%)	1.00	<0.0001	3114.9	3132.6
	A/C	295 (20.5%)	468 (38.6%)	3.99 (3.33-4.80)			
	A/A	39 (2.7%)	303 (25.0%)	19.56 (13.77-27.80)			

Dominant	C/C	1108 (76.8%)	440 (36.3%)	1.00	<0.0001	3206.3	3218.0
	A/C-A/A	334 (23.2%)	771 (63.7%)	5.81 (4.91-6.89)			

Recessive	C/C-A/C	1403 (97.3%)	908 (75.0%)	1.00	<0.0001	3343.6	3355.4
	A/A	39 (2.7%)	303 (25.0%)	12.00 (8.51-16.93)			

Overdominant	C/C-A/A	1147 (79.5%)	743 (61.4%)	1.00	<0.0001	3555.2	3567.0
	A/C	295 (20.5%)	468 (38.6%)	2.45 (2.06-2.91)			

Minor allele frequency (T)		0.13	0.44	—	—	—	—

OR: odds ratios; CI: confidence intervals; AIC: Akaike information criterion; BIC: Bayesian information criterion.

**Table 4:** Factors associated with dead patients infected with COVID-19.

Factors		
Baseline predictors	OR (95% CI)	*P* value
Mean age ± SD	0.967 (0.956-0.978)	<0.001^∗^
ALP, IU/L	0.997 (0.996–0.999)	0.003^∗^
ALT, IU/L	0.984 (0.977-0.990)	<0.001^∗^
HDL, mg/dL	1.035 (1.022-1.047)	<0.001^∗^
LDL, mg/dL	1.018 (1.015-1.022)	<0.001^∗^
FBS, mg/dL	0.996 (0.993-0.999)	0.010^∗^
Uric acid, mg/dL	1.933 (1.762-2.120)	<0.001^∗^
Creatinine, mg/dL	0.089 (0.058-0.136)	<0.001^∗^
ESR, mm/1st h	0.971 (0.962-0.980)	<0.001^∗^
CRP, mg/L	0.980 (0.974-0.987)	0.004^∗^
25-hydroxyvitamin D, ng/ml	1.040 (1.028-1.052)	<0.001^∗^
Real-time PCR Ct values	0.982 (0.963-0.998)	0.041^∗^
ABO blood groups	1.293 (1.169-1.431)	<0.001^∗^
*ABO* rs657152 (AA)	0.195 (0.155-0.247)	<0.001^∗^

ALP: alkaline phosphatase; ALT: alanine aminotransferase; HDL: high density lipoprotein; LDL: low density lipoprotein; FBS: fasting blood glucose; ESR: erythrocyte sedimentation rate; CRP: C-reactive protein; Ct: cycle threshold; SD: standard deviation. ^∗^Statistically significant (< 0.05).

## Data Availability

The data used to support the findings of this study are included within the article and supplementary file.

## Supplementary Materials

Supplementary Materials Supplementary Figure 1: The result of *ABO* rs657152 genotyping with T-ARMS-PCR. Line 4: CC genotype, Line 1 and 3: AA genotype, and Line 2: AC genotype. Supplementary Figure 2: The sequencing results of *ABO* rs657152 genotypes for confirming the T-ARMS-PCR method (10% of samples randomly were sequenced).
