# Supplementary material for: ABO rs657152 and Blood Groups Are as Predictor Factors of COVID-19 Mortality in the Iranian Population
Source: Dis Markers. 2022 Nov 14;2022:5988976. doi: 10.1155/2022/5988976 (PMC9678483; doi:10.1155/2022/5988976)
Supplement: Supplementary Materials — Supplementary Figure 1: The result of ABO rs657152 genotyping with T-ARMS-PCR. Line 4: CC genotype, Line 1 and 3: AA genotype, and Line 2: AC genotype. Supplementary Figure 2: The sequencing results of ABO rs657152 genotypes for confirming the T-ARMS-PCR method (10% of samples randomly were sequenced). [file 5988976.f1.docx]

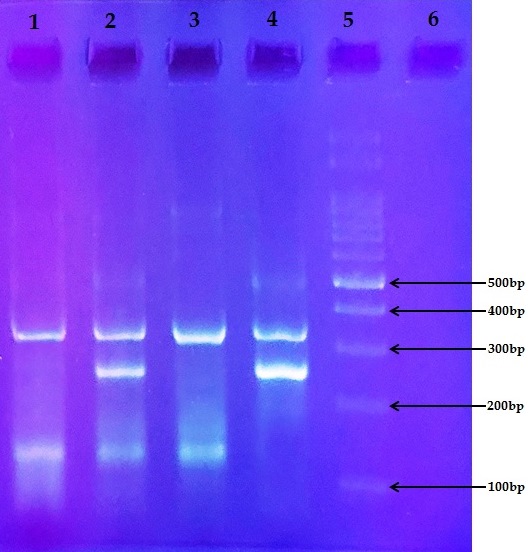


**Supplementary Figure 1:** The result of *ABO* rs657152 genotyping with T-ARMS-PCR. Line 4: CC genotype, Line 1 and 3: AA genotype, Line 2: AC genotype


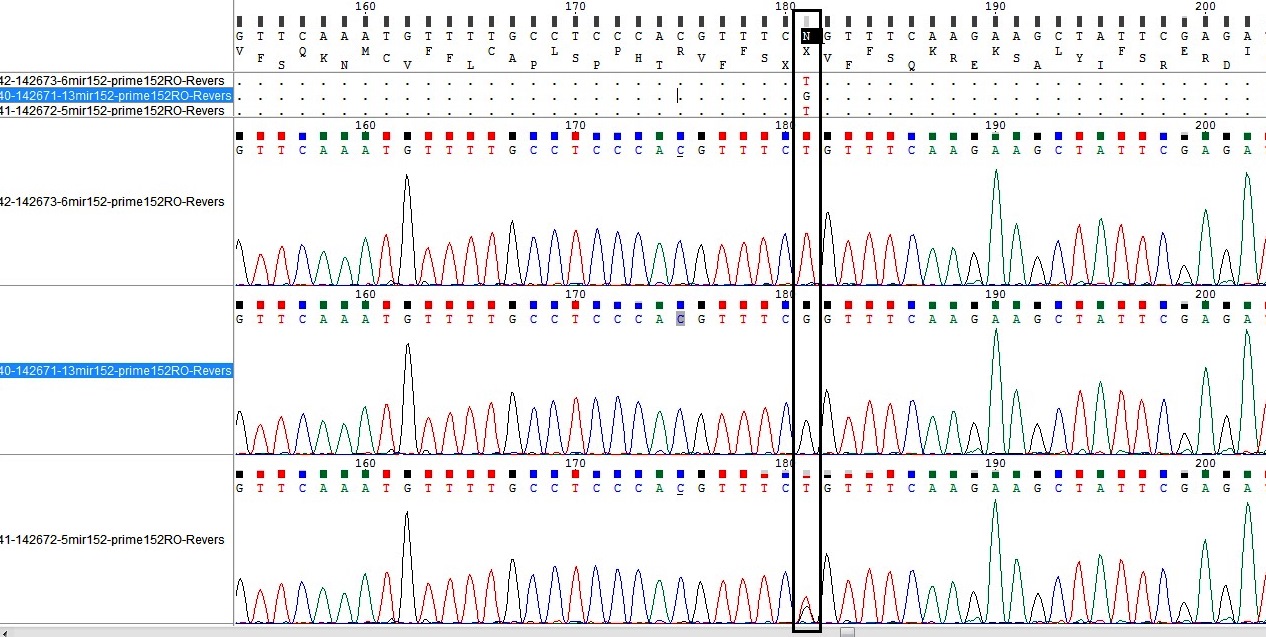


**Supplementary Figure 2:** The sequencing results of *ABO* rs657152 genotypes for confirming the T-ARMS-PCR method (10% of samples randomly were sequenced)
